# Improving catalytic activity of the Baeyer–Villiger monooxygenase-based *Escherichia coli* biocatalysts for the overproduction of (*Z*)-11-(heptanoyloxy)undec-9-enoic acid from ricinoleic acid

**DOI:** 10.1038/s41598-018-28575-8

**Published:** 2018-07-06

**Authors:** Ji-Min Woo, Eun-Yeong Jeon, Eun-Ji Seo, Joo-Hyun Seo, Dong-Yup Lee, Young Joo Yeon, Jin-Byung Park

**Affiliations:** 1 Department of Food Science and Engineering, Ewha Womans University, Seoul, 03760 Republic of Korea; 2 Department of BT-Convergent Pharmaceutical Engineering, Sun Moon University, Asan, 31460 Republic of Korea; 3 School of Chemical Engineering, Sungkyunkwan University, Suwon, 16419 Republic of Korea; 4 Department of Biochemical Engineering, Gangneung-Wonju National University, Gangneung, 25457 Republic of Korea; 5 Institute of Molecular Microbiology and Biosystems Engineering, Ewha Womans University, Seoul, 03760 Republic of Korea

## Abstract

Baeyer–Villiger monooxygenases (BVMOs) can be used for the biosynthesis of lactones and esters from ketones. However, the BVMO-based biocatalysts are not so stable under process conditions. Thereby, this study focused on enhancing stability of the BVMO-based biocatalysts. The biotransformation of ricinoleic acid into (Z)-11-(heptanoyloxy)undec-9-enoic acid by the recombinant *Escherichia coli* expressing the BVMO from *Pseudomonas putida* and an alcohol dehydrogenase from *Micrococcus luteus* was used as a model system. After thorough investigation of the key factors to influence stability of the BVMO, Cys302 was identified as an engineering target. The substitution of Cys302 to Leu enabled the engineered enzyme (i.e., E6BVMO_C302L_) to become more stable toward oxidative and thermal stresses. The catalytic activity of E6BVMO_C302L_-based *E*. *coli* biocatalysts was also greater than the E6BVMO-based biocatalysts. Another factor to influence biocatalytic performance of the BVMO-based whole-cell biocatalysts was availability of carbon and energy source during biotransformations. Glucose feeding into the reaction medium led to a marked increase of final product concentrations. Overall, the bioprocess engineering to improve metabolic stability of host cells in addition to the BVMO engineering allowed us to produce (Z)-11-(heptanoyloxy)undec-9-enoic acid to a concentration of 132 mM (41 g/L) from 150 mM ricinoleic acid within 8 h.

## Introduction

Since the Baeyer–Villiger monooxygenases (BVMOs, EC 1.14.13.x) were first isolated in 1976^1^, the enzymes have been intensively studied for oxygenation of a variety of ketone substrates^2–6^. As compared to chemical oxidations using m-chloroperoxybenzoic acid or hydrogen peroxide, the BVMO reactions have shown high chemo- and regioselectivities. However, most BVMOs were unstable under biotransformation conditions limiting their synthetic applications^7–9^. Thus, a multitude of studies have focused on the protein engineering^10–19^ and the bioprocess engineering^15,20–25^ as well as the structural properties and the reaction mechanisms^26–32^ to achieve high product concentrations with high productivities.

One of the representative examples was the BVMO from *Pseudomonas putida* KT2440^8^. The enzyme was able to catalyze the Baeyer–Villiger oxygenation of various substrates ranging from simple aliphatic linear ketones (e.g., 4-decanone) to keto-fatty acids such as 12-keto-octadec-9-enoic acid (Scheme S1), 12-keto-octadecanoic acid, 13-keto-octadec-9-enoic acid, and 10-keto-octadec-12-enoic acid^5,33–36^. Since the BVMO from *P*. *putida* KT2440 was unstable and difficult to express in a functional form in whole-cells (e.g., *Escherichia coli*, *Corynebacterium glutamicum*, and *Saccharomyces cerevisiae*), the enzyme was engineered to improve its stability and functional expression level under biotransformation conditions^14,15,37^. For instance, enzyme fusion with a soluble polyionic peptide tag (i.e., hexa-glutamate (E6))^15^ or a highly soluble enzyme (i.e., alcohol dehydrogenase of *Micrococcus luteus*)^14^ led to a significant improvement in functional expression and structural stability of the BVMO in *E*. *coli* under process conditions. By using the engineered enzyme (i.e., E6BVMO)-based *E*. *coli* biocatalyst, the ester (*Z*)-11-(heptanoyloxy)undec-9-enoic acid (**3**), which can be hydrolyzed into industrially relevant medium chain carboxylic acids (i.e., n-heptanoic acid (**4**) and 11-hydroxyundec-9-enoic acid (**5**)) (Scheme S1), could be produced to a concentration of 85 mM in the reaction medium^15^.

The major goal of the present study was to identify the factors that influence stability of the BVMO (i.e., E6BVMO) from *P*. *putida* KT2440 during *E*. *coli*-based biotransformation of fatty acids (i.e., ricinoleic acid) (Scheme S1). Another goal was to characterize the factors to improve catalytic stability of the BVMO-based *E*. *coli* biocatalysts. Ultimately, use of the newly engineered enzyme (E6BVMO_C302L_) and feeding of carbon and energy source into the reaction medium allowed us to produce the ester (**3**) to final a concentration of 132 mM (41 g/L) in the cultivation medium without applying any *in situ* product recovery system.

## Results

### Stability of E6BVMO

The engineered enzyme E6BVMO^15^, which was produced via fusion of the EthA from *P*. *putida* KT2440 with a hexa-glutamate tag on its N-terminal, was quite stable resulting in a rather high productivity of whole-cell biotransformation with ricinoleic acid (Scheme S1). However, the specific whole-cell biotransformation rate was gradually decreased over reaction times. Thus, it is imperative to identify key factors affecting the specific biotransformation rates.

**Figure 1:**
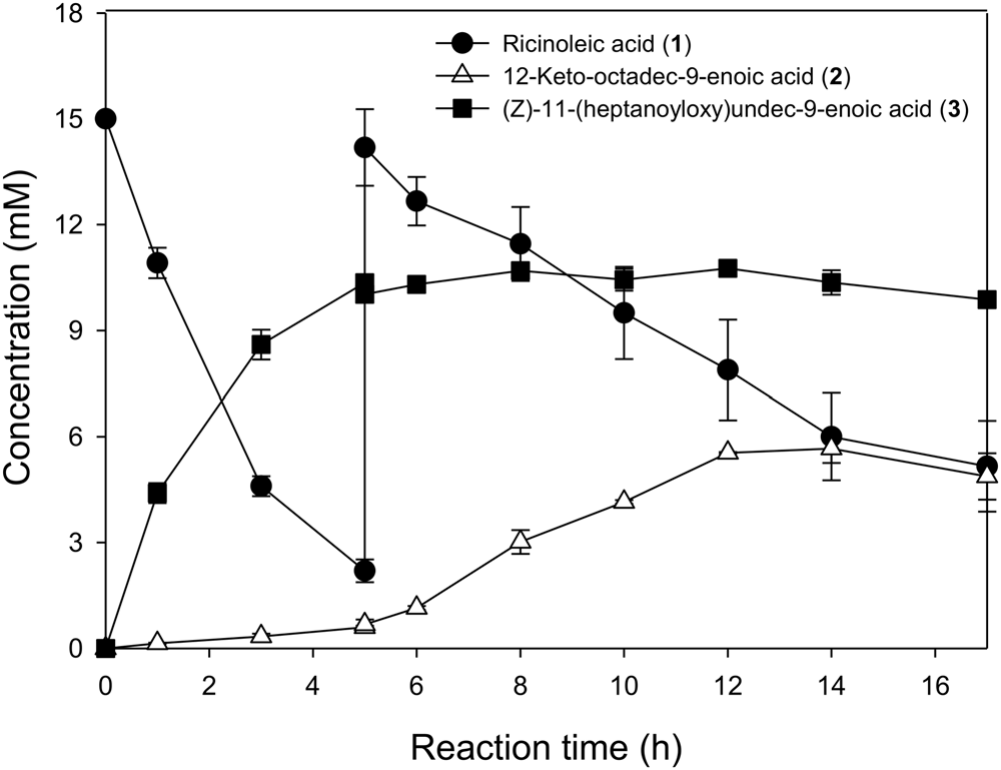
Time course of the biotransformation of ricinoleic acid by the recombinant *E*. *coli* BL21(DE3) pAPTm-E6BVMO-ADH, expressing an alcohol dehydrogenase (ADH) of *Micrococcus luteus* and the engineered Baeyer-Villiger monooxygenase (BVMO) from *Pseudomonas putida* KT2440 (i.e., E6BVMO). The biotransformation was initiated by adding 15 mM ricinoleic acid and 0.5 g/L Tween80 at t = 0 into the recombinant *E*. *coli* culture broth (cell density: 3 g cell dry weight (CDW)/L). Ricinoleic acid was added once more to a concentration of 15 mM at t = 5 h. Symbols indicate the concentrations of ricinoleic acid (**1**) (●), 12-keto-octadec-9-enoic acid (**2**) (△), and the ester **(3**) (■). The experiments were performed in triplicate. The error bars indicate standard deviations.

The biotransformation of ricinoleic acid by the recombinant *E*. *coli* BL21(DE3) pAPTm-E6BVMO-ADH, expressing an alcohol dehydrogenase (ADH) of *Micrococcus luteus* and E6BVMO, was conducted according to our previous study^15^, except for the second addition of ricinoleic acid into the cultivation medium at t = 5 h (Fig. 1). Ricinoleic acid, which was added to 15 mM at t = 0, was efficiently converted into the ester (**3**) with a conversion yield of over 80%. The reaction intermediate (**2**) concentration in the cultivation medium was below 0.5 mM until t < 5 h, indicating that the cascade enzymes were both active during the biotransformation. However, the reaction intermediate (**2**) concentration began to increase up to over 5 mM at t > 5 h while the ester (**3**) concentration remained rather unchanged. This result indicated that the catalytic activity of E6BVMO might be reduced during the biotransformation.

**Figure 2:**
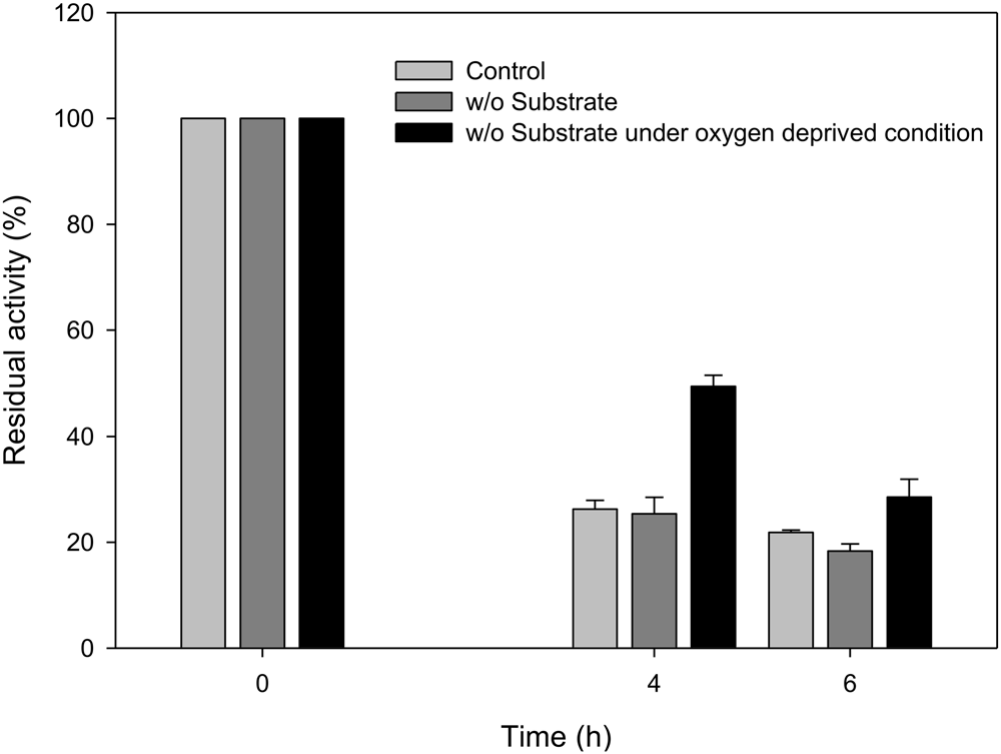
The *in vitro* activity of E6BVMO, which was prepared from the cells taken out at t = 0, 4, and 6 h during the biotransformation shown in Fig. 1. The second and the third bars indicated the *in vitro* activities of E6BVMOs, which were prepared during the biotransformations without the reaction substrate under aerobic condition and without the reaction substrate under oxygen-deprived condition, respectively. The E6BVMO activity at t = 0 was 0.056 U/mg proteins. The experiments were performed in triplicate. The error bars indicate standard deviations.

With an aim to examine the catalytic activity of E6BVMO during the whole-cell biotransformation shown in Fig. 1, the activity was estimated with *in vitro* NADPH assay (Fig. 2). Expectedly, it was reduced over the reaction times; its residual activity prepared from the cells sampled at t = 6 h was about 20% of the initial E6BVMO activity at t = 0 (i.e., 0.013 U/mg proteins). The SDS-PAGE analysis of the E6BVMO showed that the soluble form of E6BVMO was decreased with reaction time, pointing out the reduction of E6BVMO activity (Fig. S1A).

### The factors to influence E6BVMO stability

We have next investigated the factors that influence the E6BVMO activity during the whole-cell biotransformation shown in Fig. 1. The first target was the fatty acid and its derivatives, because hydrophobic compounds can be toxic to microbial cells^38–42^. The biotransformation was conducted at the condition identical to the experiment shown in Fig. 1. However, the reaction substrate (**1**) was not added into the cultivation medium to exclude any toxic effects of fatty acid and its derivatives to E6BVMO and host cell. The *in vitro* catalytic activity of the E6BVMO, which was prepared from the cells in the absence of the reaction substrate was comparable to that of the cells prepared during the biotransformation in (Figs 1 and 2). This result indicated that ricinoleic acid (**1**) and its derivatives (**2** and **3**) would not be responsible for the decrease of E6BVMO activity during the biotransformation.

The *in vitro* catalytic activity of the E6BVMO, which was prepared from the cells incubated under the biotransformation condition but without ricinoleic acid under oxygen-deprived condition, was measured (Fig. 2). The residual activity of E6BVMO at the cells taken out at t = 4 h was significantly greater than that of E6BVMO at the control cells (i.e., 0.025 U/mg proteins). This result was also supported by the higher level of E6BVMO in the soluble fraction of cell extracts as shown in the SDS-PAGE analysis (Fig. S1A and C). Overall, it was assumed that oxygen-mediated toxic metabolites (e.g., reactive oxygen species (ROS), which can be produced from a flavin-dependent monooxygenase BVMO^43–46^) could be involved in the decrease of E6BVMO activity during the whole-cell biotransformation.

### Identification of engineering targets

The BVMOs were reported to be susceptible to oxidative stress^16^. In more specific, cysteine and methionine containing sulfur group can be highly sensitive to the ROS. Cysteine may produce sulphenic acid intermediates and sulfinic acid by hydrogen peroxide and other oxidants^47,48^. The BVMO from *P*. *putida* KT2440 contains 12 Met and 8 Cys residues (Fig. S2A). Among them, Cys302 is positioned within ca. 8.7 Å from the flavin peroxide (Fig. S2B) in the active site, which may generate the ROS^43–46^. Thereby, Cys302 was suggested as an engineering target.

### Construction and characterization of an E6BVMO mutant

**Figure 3:**
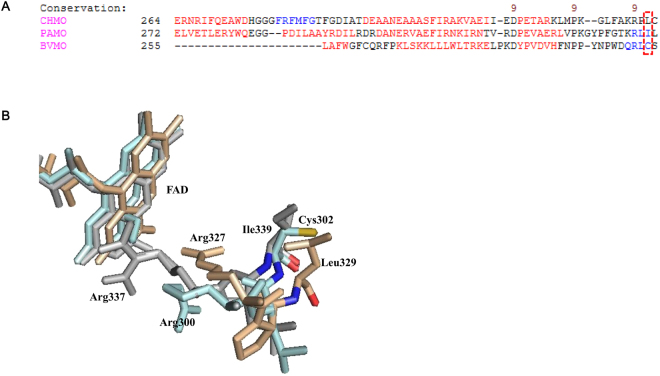
The sequence (**A**) and the three-dimensional structure (**B**) alignments of the BVMO of *P*. *putida* KT2440 with the phenyl acetone monooxygenase (PAMO) from *Thermobifida fusca* and the cyclohexanone monooxygenase (CHMO) from *Rhodococcus* sp. strain HI-31. The previously reported homology model of the *P*. *putida* KT2440 BVMO^15^ and the crystal structures of the PAMO (PDB code 1W4X) and the CHMO (PDB code 3GWD and 3GWF) were used for the study. The cyan, grey and yellow colors indicated the structure of *P*. *putida* KT2440 BVMO, PAMO, and CHMO, respectively.

It was difficult to find the conserved region around Cys302 via the sequence alignments of E6BVMO with the phenyl acetone monooxygenase (PAMO) from *Thermobifida fusca* and the cyclohexanone monooxygenase (CHMO) from *Rhodococcus* sp. strain HI-31, which are known to be relatively stable (Fig. 3A). On the other hand, alignments of the homology model of E6BVMO^15^ with the structural models of PAMO (PDB code 1W4X) and CHMO (PDB code 3GWD and 3GWF) suggested that the enzymes have Ile or Leu that are nonoxidizable hydrophobic in the corresponding site (Fig. 3B). Therefore, the point mutation of Cys302 into Leu was investigated.

**Figure 4:**
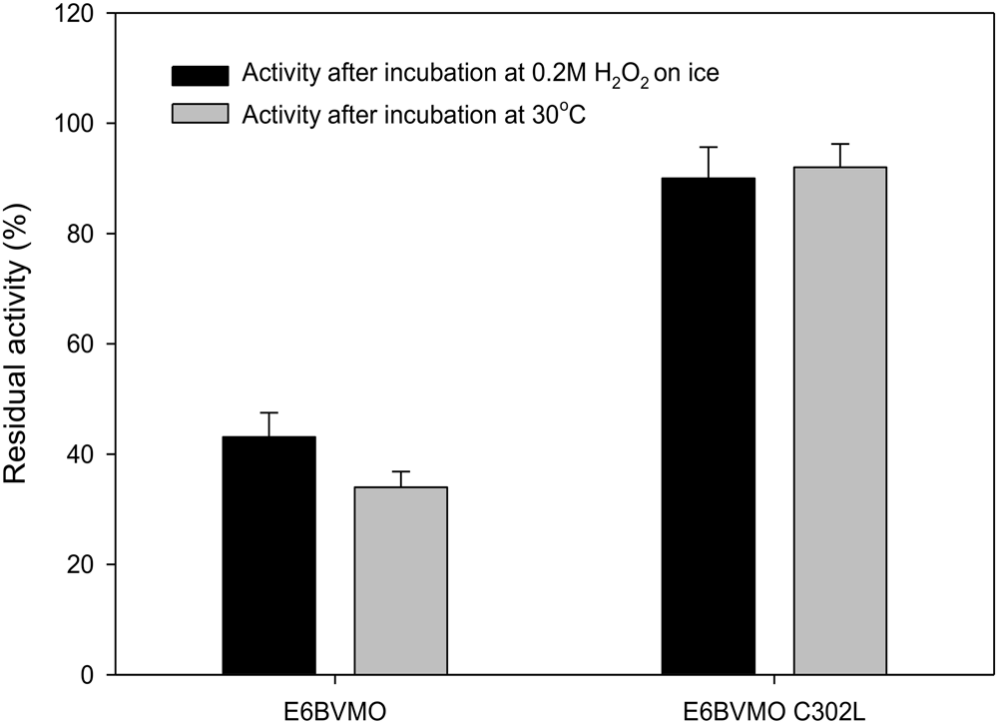
The stabilities of E6BVMO and E6BVMO_C302L_ to oxidative and thermal stress. The oxidative stabilities of the enzymes (black bars) were measured 10 min after incubation at the Tris-HCl buffer (pH 8) containing 0.2 M hydrogen peroxide on ice. The thermal stabilities of the enzymes (grey bars) were evaluated 10 min after incubation at the Tris-HCl buffer at 30 °C. The enzymes, which were purified via affinity chromatography on a Ni-NTA gel matrix, were used. The initial activity of E6BVMO and E6BVMO_C302L_ was 0.9 and 1.0 U/mg proteins, respectively. The experiments were performed in triplicate. The error bars indicate standard deviations.

After construction of E6BVMO_C302L_ by site-directed mutagenesis, the recombinant plasmid (pET-E6BVMO_C302L_ (Table S1)) was transformed into *E*. *coli* BL21(DE3). The engineered enzyme E6BVMO_C302L_ was purified via affinity chromatography on a Ni-NTA gel matrix after expression at 16 °C. Subsequently, the purified enzyme was subjected to stability assay against oxidative and thermal stress (see the Materials and Methods for details). When the residual activity was measured after 10 min incubation with 0.2 M H_2_O_2_, the engineered BVMO (E6BVMO_C302L_) exhibited much higher activity as compared to the control enzyme E6BVMO (Fig. 4); the control enzyme has lost its activity by over 50% after incubation in the presence of hydrogen peroxide, but the engineered enzyme was able to keep at least 90% activity (i.e., 0.9 U/mg proteins). This result indicated that the Cys302 was one of the key amino acid residues, which are susceptible to the oxidative stress.

The newly engineered E6BVMO_C302L_ has also shown the greater residual activity after incubation at a room temperature (i.e., 30 °C) for 10 min (Fig. 4). Furthermore, the half-life of E6BVMO_C302L_, the time at which 50% catalytic activity was left, was increased approximately ten folds to 60 min at 30 °C, as compared to that of E6BVMO (Fig. S3A). The difference in catalytic stability at 4 °C was much more significant (Fig. S3B). These results indicated that the substitution of Cys302 to Leu conferred a thermal stability on the enzyme. Overall, it was assumed that stability of the E6BVMO against not only oxidative but also thermal stress was improved by the C302L mutation in the active site.

With an aim to investigate effect of C302L on the reaction kinetics of E6BVMO, the kinetic constants were determined (Fig. S4). The *k*_*cat*_ and *K*_*m*_ values of E6BVMO_C302L_were comparable to those of E6BVMO. This result indicated that the C302L mutation did not have a marked effect on the catalytic activity of E6BVMO.

### Stability of E6BVMO_C302L_ under biotransformation conditions

**Figure 5:**
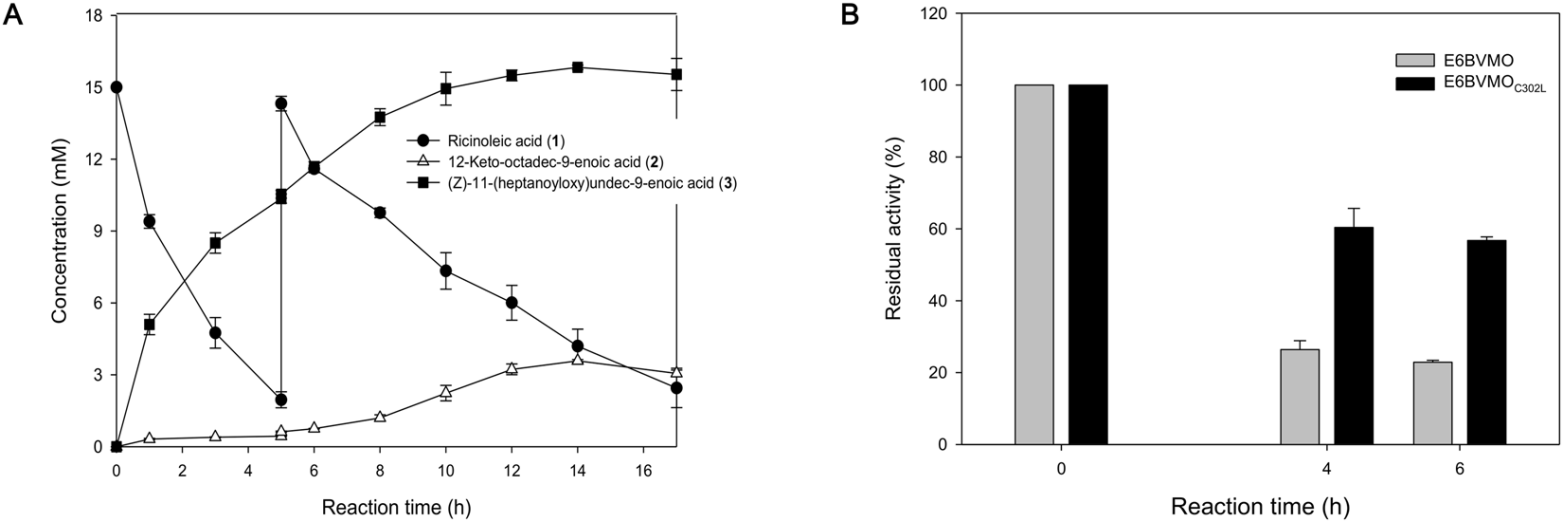
Time course of the biotransformation of ricinoleic acid by the recombinant *E*. *coli* BL21(DE3) pAPTm-E6BVMO_C302L_-ADH, expressing the ADH of *M*. *luteus* and the engineered E6BVMO (i.e., E6BVMO_C302L_) (**A**) and the *in vitro* activity of E6BVMO_C302L_, which was prepared from the cells at t = 0, 4, and 6 h during the biotransformation shown in (**A,B**). The whole-cell biotransformation was conducted at the same condition as the biotransformation shown in Fig. 1. The *in vitro* activity of E6BVMO_C302L_ at t = 0 was 0.050 U/mg proteins. Symbols indicate the concentrations of ricinoleic acid (**1**) (●), 12-keto-octadec-9-enoic acid (**2**) (△), and the ester (**3)** (■). The experiments were performed in triplicate. The error bars indicate standard deviations.

The biotransformation of ricinoleic acid by the recombinant *E*. *coli* BL21(DE3) pAPTm-E6BVMO_C302L_-ADH (Table S1), expressing the ADH of *M*. *luteus* and the E6BVMO_C302L_, was conducted at the condition identical to the experiment shown in Fig. 1. The biotransformation profiles at t < 5 h were comparable whereas the BVMO reaction product ester (**3**) was continuously produced even after second feeding of ricinoleic acid into the cultivation medium (Fig. 5A). The ester (**3**) concentration reached over 15 mM in the medium.

The *in vitro* activity of E6BVMO_C302L_ (Fig. 5B), which was prepared from the cells at t = 4 and 6 h, was ca. three-fold higher than that of E6BVMO prepared at the comparable condition shown in Fig. 1. Therefore, it was concluded that E6BVMO_C302L_ is also more stable under biotransformation condition as compared to E6BVMO.

### Further engineering of E6BVMO_C302L_

The E6BVMO_C302L_ was further engineered to improve its thermal stability. The engineering targets were selected by using the protein thermal stability prediction program, TargetStar^49,50^. In total, 23 double mutants (Table S1) were chosen and 10 of them (C302L-K483I, C302L-K483A, C302L-D179I, C302L-D179F, C302L-R327I, C302L-R327V, C302L-R327L, C302L-R327F, C302L-R327W and C302L-R327Y) were predicted to have a higher thermal stability by SDM (Site Directed Mutator)^51^ software too. After construction of the mutants by site-directed mutagenesis, biocatalytic activities of the E6BVMO_C302L_-based double mutants were first evaluated via whole-cell biotransformation of ricinoleic acid (Fig. S5). Most of the mutants showed lower whole-cell biotransformation activities as compared to the E6BVMO_C302L_, while the three mutants (i.e., C302L-R327I, C302L-R327A, C302L-R327M) showed comparable activities. The thermal and oxidative stabilities of the five mutants including C302L-R327I, C302L-R327A, C302L-R327M were investigated after purification (Fig. S6). Although the oxidative stabilities of the mutants were comparable to the E6BVMO_C302L_, the thermal stabilities remained lower except for C302L-K483A.

### Engineering of the E6BVMO_C302L_-based *E*. *coli* biocatalyst

The nicotinamide cofactors are involved at the first and second biotransformation steps (Scheme S1). NAD^+^ and NADPH are served as electron acceptor and donor, respectively. Probably, NADH, which is generated at the first step, could be used for regeneration of NADPH by transhydrogenases in *E*. *coli*^52^. In order to facilitate the NADPH regeneration, the PntAB, which was shown to enhance availability of NADPH in bacterial cells (e.g., *E*. *coli* and *Corynebacterium glutamicum*)^53,54^ was overexpressed in the recombinant *E*. *coli* BL21(DE3) expressing the E6BVMO_C302L_ and ADH. However, the biotransformation activity of the resulting recombinant *E*. *coli* strain was not greater as compared to that of the control strain.

As another approach to improve regeneration of the nicotinamide cofactors, glucose was added to 5 g/L into the culture medium just after the second addition of ricinoleic acid into the cultivation medium at t = 5 h. The final product concentration was increased up to 17 mM in the reaction medium, indicating that glucose might be used as an energy source during the biotransformation.

### Productivity of E6BVMO_C302L_-based whole-cell biotransformation process

**Figure 6:**
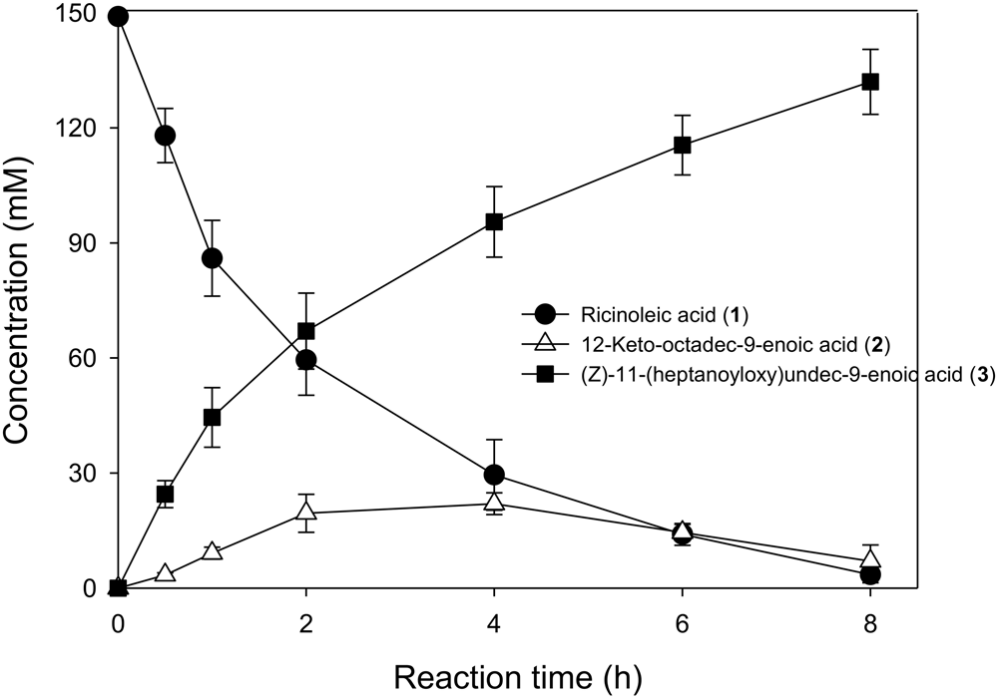
Time course of the biotransformation of ricinoleic acid by the recombinant *E*. *coli* BL21(DE3) pAPTm- E6BVMO_C302L_-ADH, pACYC-FadL expressing the long chain fatty acid transporter FadL in the outer membrane in addition to the ADH and E6BVMO_C302L_. The biotransformation was initiated by adding 150 mM ricinoleic acid and 0.5 g/L Tween80 into the recombinant *E*. *coli* culture broth (cell density: 25 g CDW/L). Glucose was added into the cultivation medium to a rate of 5 g/L/h to facilitate regeneration of the nicotinamide cofactors in *E*. *coli* cells. Symbols indicate the concentrations of ricinoleic acid (**1**) (●), 12-keto-octadec-9-enoic acid (**2**) (△), and the ester **(3)** (■). The experiments were performed in triplicate. The error bars indicate standard deviations.

The biotransformation performance of E6BVMO_C302L_-based biocatalyst (i.e., the recombinant *E*. *coli* BL21(DE3) pAPTm-E6BVMO_C302L_-ADH, pACYC-FadL) was then evaluated in a bioreactor. After high cell density cultivation of the recombinant strain to 25 g CDW/L, ricinoleic acid was added to a concentration of 150 mM in the cultivation medium with 0.5 g/L Tween80 (see the Materials and Method for details). Glucose was added into the cultivation medium to a rate of 5 g/L/h to facilitate regeneration of the nicotinamide cofactors in *E*. *coli* cells. The target product (**3**) accumulated up to 132 mM (41 g/L) in the cultivation medium at t = 8 h with an average biotransformation rate of 16 mM/h (Fig. 6). The concentration of the reaction intermediate (**2**) remained below 15 mM in the reaction medium, indicating that E6BVMO_C302L_ was active throughout the biotransformation. The product yield, which was calculated on a basis of the ester concentration, determined by gas chromatography/mass spectrometry (GC/MS), reached ca. 88%. The crude ester products isolated via extraction with ethyl acetate and evaporation *in vacuo* were subjected to hydrogenation of the C9 double bond, hydrolysis of the ester bond, and oxidation of the terminal hydroxyl group to carboxylic acid, as reported previously^23^. The conversion yield of the three chemical steps to produce 1,11-undecanedioic acid from the ester was over 80%. Ultimately, 25 g of 1,11-undecanedioic acid was isolated and recovered from 1 L of reaction medium in addition to 15 g of heptanoic acid (overall molar isolation yield of 1,11-undecanedioic acid from ricinoleic acid: 68%).

As compared to the E6BVMO-based whole-cell biotransformation under comparable conditions (Table 1 and Fig. S7), the engineered enzyme (E6BVMO_C302L_)-based *E*. *coli* biocatalysis allowed approximately 40% greater final product concentration with similar initial specific biotransformation rate. This result indicated that stability of E6BVMO_C302L_ played a key role in the whole-cell biotransformation of ricinoleic acid into the ester (**3**), in particular, in the final product concentration.

**Table 1:** Biocatalytic performance of the E6BVMO_C302L_-based biocatalyst in a bioreactor.

	Biotransformation 1^a^	Biotransformation 2^a^
Biocatalyst	*E*. *coli* BL21(DE3) pAPTm- E6BVMO_C302L_-ADH, pACYC-FadL	*E*. *coli* BL21(DE3) pAPTm- E6BVMO-ADH, pACYC-FadL
Ricinoleic acid concentration (mM)	150	150
Biocatalyst concentration (g CDW/L)	25	25
Final product (**3**) concentration (mM)	128 ± 5	92
Volumetric productivity (mM/h)^b^	16 ± 0.7 (32 ± 2)	12 (29)
Product yield (%)^c^	85 ± 4	61

^a^Biotransformation 1 and 2 indicates the experiment shown in Figs 6 and S7, respectively.

^b^Volumetric productivity was calculated based on the ester product concentration, which was determined by GC/MS, and the reaction time, which was measured when >90% of the starting material was converted to the products. The values in parenthesis indicate initial biotransformation rates, which were measured at t < 2 h.

^c^Product yield was calculated based on the initial substrate concentration and the final ester product concentration, which were determined by GC/MS.

## Discussion

BVMOs are capable of catalyzing a variety of Baeyer-Villiger oxygenations with high regioselectivities and thereby considered as one of the important biocatalysts for organic synthesis^2–6^. However, most BVMOs are rather unstable under reaction conditions^7–9^. One of the reasons may include generation of the ROS during biocatalysis^46,55^. The ROS may lead to oxidation of cysteine to sulfenic acid, sulfinic acid and cysteine thiyl radicals, which may in turn result in protein damage and degradation^47,48,55^. A number of studies have reported stabilization of the enzymes against oxidative stress^16,17,25,56–59^. One example was engineering the CHMO from *Acinetobacter* sp. NCIMB 9871^16^. The enzyme stability was substantially improved by multiple mutations including M5I, M291I, C330S, C376L, M412L, M481A, and C520V. Another reason for the low stability of BVMOs would be the large conformational change during catalysis^27^. The great stability of the PAMO from *T*. *fusca* and the CHMO from *Thermocrispum municipale*^28^ was suggested with a high number of salt bridges and hydrogen bonds compared to other BVMOs^60^. Our previous study^15^ also showed that structural stability of the BVMO from *P*. *putida* KT2440 under high temperature was improved via generation of salt-bridge between Glu residues of the E6 tag and the Arg residue sitting around N-terminal of the BVMO.

In the present study, substitution of Cys302 in the active site to Leu resulted in a significant increase of BVMO stability to oxidative and thermal stress (Fig. 4). According to the structure modeling study, C302L appeared to attenuate oxidation of the active site by replacing the Cys residue, which is susceptible to oxidative stress, with a rather inert amino acid residue (e.g., Leu) (Fig. S2). Furthermore, the C302L mutation seemed to enhance the hydrophobic interactions with proximal Ala194 and Val197 in *α*-helical structure (Fig. S8). The hydrophobic interactions may increase rigidity of the active site and thus structural stability of the engineered enzyme. Another reason for the higher thermostability may include the increased tolerance of E6BVMO_C302L_ to oxidative stress, because oxidative stress was reported to be enhanced under high temperatures^16^. Overall, the great stability of E6BVMO_C302L_ to oxidative and thermal stress appeared to be achieved with increase in rigidity of the active site as well as with increase in tolerance to oxidative stress.

A similar study to improve oxidative and thermal stabilities of the CHMO from *Acinetobacter* sp. NCIMB 9871 was previously reported^16^. Remarkably, mutation of C330L in the CHMO, which corresponds to C302L in E6BVMO (Fig. S9A), resulted in a significant decrease of oxidative and thermal stabilities. The C330L mutation appeared to inhibit soluble expression of CHMO_C330L_ and let the enzyme structurally unstable. According to the structural models, Leu302 of E6BVMO_C302L_ is surrounded by hydrophobic amino acid residues such as Ala194 and Val197 (Fig. S8), whereas vicinity of Leu330 of CHMO_C330L_ is not so hydrophobic (Fig. S9B). Asp56, Tyr80 and Ile215 are located nearby Leu330. Therefore, it was assumed that microenvironment of the cysteine/leucine targeted is important to acquire tolerance to oxidative and thermal stress.

We have also studied thermal stability of E6BVMO_C302L_ by using TargetStar and SDM programs^49–51^. The structural features of the five selected mutants (C302L, C302L-R327I, C302L-R327L, C302L-R327M, C302L-R327A) were examined by investigating the intramolecular interactions of the variants via *in silico* mutations and free energy minimization using Maestro program of Schrödinger software package^61–63^ (Table S3 and Fig. S10). E6BVMO_C302L_ was found to have a salt bridge between R327 and E330, and three hydrogen bonds (R327-E330, R327-Q165, E330(SO)-E330(MN)) in the vicinity of the ionic bond, while no aromatic hydrogen bonds, pi-pi stacking and pi-cation interactions were detected. The salt bridge and two hydrogen bonds (R327-E330 and R327-Q165) in E6BVMO_C302L_ were removed when additional four mutations (R327I, R327L, R327M, R327A) were applied. Although C302L-R327I, C302L-R327L variants were predicted to have enhanced stability by TargetStar and SDM program both via increased ΔΔG values and newly formed hydrogen bonds (E330-H331 and/or H331-K342), they showed decreased thermal stability compared to the E6BVMO_C302L_. The local ionic bond breakage between R327 and E330 might be ascribed to the adverse effects on the stability of the variants than the overall energy improvements in the protein structure. On the other hand, the comparable thermal stability of C302L-K483A variant compared to the E6BVMO_C302L_ might be due to the minor structural changes in one hydrogen bond (K483-Y377) (Fig. S11).

This study also showed that amount of the target product (**3**) produced per g biocatalysts were around 40% higher with E6BVMO_C302L_ as compared to E6BVMO (Table 1). This result indicated that the stability of E6BVMO_C302L_ was greater not only *in vitro* but also *in vivo* environments. The significantly higher final product concentration with E6BVMO_C302L_ has suggested that stability of the cascade enzymes is one of the key factors to determine final product concentration in whole-cell biocatalysis. The catalytic performance of *E*. *coli*-based biocatalysts was also influenced by glucose feeding during biotransformations. Since glucose could be used as a carbon and energy source, which is required for nicotinamide cofactor regeneration and protein turnover, bioprocess engineering to improve metabolic stability of host cells might be another key factor.

## Conclusion

This study has demonstrated that the oxidative stress was one of the main factors to affect stability of the BVMO from *P*. *putida* KT2440 during whole-cell biotransformation of fatty acids. Thereby, the C302L mutation resulted in the enhancement of tolerance against oxidative stress. Moreover, the C302L led to increase in rigidity of the active site via enhancing the hydrophobic interactions, resulting in an increase of thermal stability. Overall, the stabilized enzyme (E6BVMO_C302L_)-based *E*. *coli* biocatalyst allowed us to produce the ester (**3**) to a concentration of 132 mM (41 g/L) in the aqueous cultivation medium. This study would contribute to industrial application of BVMO-based whole-cell biocatalysis for the production of medium chain fatty acids from long chain fatty acids.

## Materials and Methods

### Microbial strains, culture conditions and expression of heterologous genes

The recombinant *E*. *coli* BL21(DE3) strains, expressing the cascade enzymes including the engineered Baeyer-Villiger monooxygenases (i.e., E6BVMO and E6BVMO_C302L_) of *P*. *putida* KT2440 (Scheme S1) were cultivated overnight in the lysogeny broth (LB) medium supplemented with appropriate antibiotics (Table S1) for seed cultivation^64^. The Riesenberg medium supplemented with 10 g/L glucose and the appropriate antibiotics was used for the main cultivation and biotransformation. The recombinant *E*. *coli* cultures were incubated at 30 °C with shaking at 250 rpm (Jeiotech, Daejeon, Korea). The cascade enzymes were constitutively expressed by a constitutive synthetic promoter (i.e., J23100 (Identifier: BBa_J23100 (http://parts.igem.org/Promoters/Catalog/Anderson)) in the pAPTm-vectors. The recombinant genes in the pACYC- and the pETDuet-vectors were expressed by adding 0.1 mM isopropyl β-D-1-thiogalactopyranoside (IPTG) into the cultivation medium.

### Reagents

Ricinoleic acid and palmitic acid were purchased from Tokyo Chemical Industry Co (Tokyo, Japan). Oleic acid, linoleic acid, and ethyl acetate were purchased from Duksan Pure Chemical Co. (Ansan, Republic of Korea). Glucose was purchased from Junsei Chemical Co (Tokyo, Japan). Antibiotics, trace elements for culture medium, and Tween80 were purchased from Sigma (St. Louis, MO, USA). *N*-Methyl-*N*-(trimethylsilyl)trifluoroacetamide (TMS) was obtained from Tokyo Chemical Industry Co. (Tokyo, Japan).

### *In vitro* BVMO activity assay

The *in vitro* BVMO activity was measured by monitoring the NADPH consumption at 340 nm for 180 s in 1 mL cuvettes by using a spectrophotometer (Thermo Fisher Scientific, MA, USA)^65^. The assays were performed in 0.1 M Tris-HCl buffer (pH 8.0) containing 0.4 mM NADPH, 10 mM 4-decanone, and appropriate amount of the crude enzyme extracts. One unit (U) of the enzyme activity was defined as the amount of enzyme to oxidize 1 μmol of NADPH for 1 min under the reaction condition.

### Site-directed mutagenesis

The site-directed mutagenesis of E6BVMO was performed by PCR using KOD Xtreme Hot Start DNA Polymerase (Novagen), according to manufacturer’s instructions. Briefly, the PCR reaction mixtures (50 μL) consisted of 2x Xtreme Buffer (25 μL), deoxynucleoside triphosphates (0.4 mM each), KOD Xtreme^TM^ Hot Start DNA Polymerase (1 U), plasmid DNA (10 ng), and both sense and anti-sense primers (10 μM) (Table S2). The PCR was started with an initial denaturation step at 94 °C for 2 min, followed by 25 cycles of denaturing at 98 °C for 10 s, annealing at 60 °C for 30 s, and extension at 68 °C for 7 min, with a final extension at 68 °C for 10 min. After the PCR, the resulting products were digested with DpnI (New England Biolabs, 0.4 U) at 37 °C for 2 h, to ensure removal of the template plasmid DNA. After the DpnI digest and gel purification (Elpis biotech), the products were treated with T4 polynucleotide kinase (Elpis biotech) to phosphorylate the 5′-ends at 37 °C for 30 min. Afterward, DNA fragments were ligated using T4 ligase (Elpis biotech) at 37 °C for 30 min, followed by transformation into *E*. *coli* DH5α. After overnight cultivation on agar medium, random colonies were selected and mutagenesis was confirmed through sequencing.

### Purification of engineered BVMOs

The recombinant *E*. *coli* BL21(DE3) expressing the engineered BVMOs (i.g., E6BVMO and E6BVMO_C302L_) were cultivated in the Riesenberg medium, after which they were harvested through centrifugation at 5,000 g for 15 min and washed with Tris-HCl (pH 8, 50 mM). The washed cells were resuspended into the Tris-HCl buffer and subjected to the cell lysis by sonication. Afterwards, the resulting enzymes were purified via affinity chromatography on a Ni-NTA gel matrix (Qiagen, Crawley, United Kingdom). A column containing 3 mL of Ni-NTA resin was equilibrated with 15 volumes of buffer (20 mM Tris, 500 mM NaCl, and 5 mM imidazole), and the supernatant was loaded onto the column. The column was washed with 10 volumes of washing buffer (20 mM Tris pH 8.0, 500 mM NaCl, and 20 mM imidazole). The target proteins were then eluted by increasing the imidazole concentration to 0.3 M. Fractions containing the recombinant proteins were pooled and dialyzed to remove imidazole.

### The BVMO stability to oxidative and thermal stress

The oxidative stabilities of the engineered BVMO enzymes were evaluated by incubating the enzymes (0.2 mg/mL) in 0.1 M Tris-HCl buffer (pH 8) containing 200 mM hydrogen peroxide on ice for 10 min, as previously described^16^. The initial and residual enzyme activities were measured by NADPH assay in the presence of 4-decanone as the substrate. One unit (U) of the enzyme activity was defined as the amount of enzyme to oxidize 1 μmol of NADPH for 1 min under the reaction condition.

The thermal stabilities of the engineered BVMO enzymes were evaluated by incubating the purified enzymes (0.2 mg/mL) in the Tris-HCl buffer at 30 °C for 10 min, as previously reported^9,16^. The initial and residual enzyme activities were measured by NADPH assay in the presence of 4-decanone as the substrate.

### Whole cell biotransformations

The whole-cell biotransformation of fatty acids (e.g., ricinoleic acid) by the recombinant *E*. *coli* in a 250 mL flask (working volume: 20 mL) was carried out on a basis of our previous studies^23,34,66^. Shortly, the biotransformation was initiated by adding 5 to 15 mM fatty acids and 0.5 g/L tween80 into the cultivation medium of the recombinant *E*. *coli* at the stationary growth phase (cell density: 3 g cell dry weight (CDW)/L). The reaction pH and temperature was set to pH 8.0 and 35 °C, respectively.

The biotransformation in a 5 L bioreactor (working volume: 3 L) was carried out based on our previous study with some modifications^15^. The recombinant *E*. *coli* BL21(DE3) pAPTm-E6BVMO_C302L_-ADH, pACYC-FadL was grown batch-wise at 30 °C until glucose (20 g/L) was exhausted. Afterwards, a mixture of glucose (600 g/L) and MgSO_4_·7H_2_O (20 g/L) was fed using the pH-stat feeding strategy. Cultivation pH was automatically maintained at pH 7.2 by feeding 28% ammonia solution. Agitation speed and aeration rate were set to 800 rpm and 1vvm, respectively, during cultivation. When the cell density has reached 25 g CDW /L, the biotransformation was initiated by adding 150 mM ricinoleic acid and 0.5 g/L Tween80 into the culture broth. Glucose was added into the cultivation medium to a rate of 5 g/L/h to facilitate regeneration of the nicotinamide cofactors in *E*. *coli* cells. The reaction pH and temperature was maintained at pH 8.0 and 35 °C, respectively. The agitation speed and aeration rate were controlled to avoid oxygen limitation in the reaction medium.

### Product analysis by GC/MS

Concentrations of fatty acid substrates, intermediates, and final products were measured as described previously^23,24,34^. The reaction medium was mixed with a twice volume of ethyl acetate containing palmitic acid as an internal standard. The organic phase was harvested after vigorous vortexing and was then subjected to derivatization with N-methyl-N-(trimethylsilyl) trifluoroacetamide (TMS). The TMS derivatives were analyzed by GC/MS (Agilent, Santa Clara, CA, USA) equipped with a flame ionization detector and a split injection system (split ratio set at 1:10) and fitted with a nonpolar capillary column (30 m length, 0.25-μm thickness, HP-5MS, Agilent Technologies, Palo Alto, CA, USA). Column temperature was increased from 90 to 255 °C at a rate of 5 °C/min, and then maintained at 255 °C. The injector and detector temperatures were 260 and 250 °C, respectively. Mass spectra were obtained by electron impact ionization at 70 eV. Scan spectra were obtained within the range of 100–600 m/z. Selected ion monitoring (SIM) was used for the detection and fragmentation analysis of the reaction products.

### Homology modeling of the engineered BVMOs

Homology model of the engineered BVMOs, which was published in our previous study^15^, was used. The model was constructed as follows; the template was chosen with CRFalign, which utilizes probabilistic pairwise alignment sequence and structure with boosted regression trees as a score function. 3UOV A chain (sequence identity: 22.1%), 3UCL A chain (sequence identity: 19.5%), 1W4X A chain (sequence identity: 18.8%), and 4AOS A chain (sequence identity: 18.7%) were used as the templates. Tertiary structures were constructed by using Modeller and structure with the lowest energy was searched by using conformational space annealing. Side chain remodeling was performed using RotamerCSA with residue-dependent rotamer library and standard SCWRL4 rotamer library^15^.

Homology model of the CHMO from *Acinetobacter* sp. NCIMB 9871^16^, which was kindly donated by Prof. Opperman, was used for the three-dimensional structure alignments of the BVMO of *P*. *putida* KT2440 with the CHMO from *Acinetobacter* sp. NCIMB 9871. In addition, the model was used to examine microenvironment of C330 in the active cite of CHMO_C330L_.

### *In silico* design of thermostable BVMOs

The prediction program TargetStar^49,50^ was used to design of thermostable BVMO. To predict the thermal stability of a given amino acid sequence (i.e., E6BVMO_C302L_), TargetStar uses energy functions and energy parameters, which consist of 20 types of amino acids, secondary structures and solvent exposed ratios established in previous studies. Ultimately, using energy functions, the thermodynamic stability score, folding transition temperature (*T*_*f*_) of the protein was calculated, and mutations with higher *T*_*f*_ value than template were selected. Next, effects of the selected mutations on the protein stability were further predicted by SDM (Site Directed Mutator) software^51^. SDM is based on knowledge of observed substitutions within homologous protein families to calculate protein stability changes (ΔΔG, kcal/mol) between a wild type and mutant protein.

### *In silico* mutation and analysis of intramolecular interactions

All mutant structures were prepared by *in silico* mutations based on the homology modeled BVMO structure in the Maestro visualization tool. All the structures were refined using Protein Preparation Wizard^67^. The correct bond orders were assigned, hydrogens were added, and waters beyond 5 Å of ligands were removed. Hydrogen bonds were optimized based on Epik^68^ calculation for proper pKa values, followed by restrained minimization using OPLS3 forcefield. All the generated structures were further minimized by Prime^69^, a side chain prediction tool. The resulting structures of mutants were analyzed for intramolecular interactions including hydrogen bonds, aromatic hydrogen bonds, salt bridges, pi-pi stacking and pi-cation interactions by Maestro program.

### Data availability

All data generated or analyzed during this study are included in this published article (and its Supplementary Information files).

## Electronic supplementary material

Supplementary information
