# Supplementary material for: Improving catalytic activity of the Baeyer–Villiger monooxygenase-based Escherichia coli biocatalysts for the overproduction of (Z)-11-(heptanoyloxy)undec-9-enoic acid from ricinoleic acid
Source: Sci Rep. 2018 Jul 6;8:10280. doi: 10.1038/s41598-018-28575-8 (PMC6035261; doi:10.1038/s41598-018-28575-8)
Supplement: Supplementary file 1 — Supplementary information [file 41598_2018_28575_MOESM1_ESM.pdf]

Supplementary Information (SI)

Improving catalytic activity of the Baeyer–Villiger monooxygenase-based *Escherichia coli* biocatalysts for the overproduction of (*Z*)-11-(heptanoyloxy)undec-9-enoic acid from ricinoleic acid

Ji-Min Woo[1], Eun-Yeong Jeon[1], Eun-Ji Seo[1], Joo-Hyun Seo[2], Dong-Yup Lee[3], Young Joo Yeon[4] and Jin-Byung Park[1,5*]

[1]Department of Food Science and Engineering, Ewha Womans University, Seoul 03760, Republic of Korea

[2]Department of BT-Convergent Pharmaceutical Engineering, Sun Moon University, Asan 31460, Republic of Korea

[3]School of Chemical Engineering, Sungkyunkwan University, Suwon 16419, Republic of Korea

[4]Department of Biochemical Engineering, Gangneung-Wonju National University, Gangneung 25457, Republic of Korea

[5]Institute of Molecular Microbiology and Biosystems Engineering, Ewha Womans University, Seoul 03760, Republic of Korea

Table S1. Plasmids used in this study

Plasmids	Features	Description	Reference
pACYC-ADH	Carrying the ADH gene of *M. luteus*	P15A origin Chlor^R, P_{T7}	Song *et al.*[1]
pETDuet-E6BVMO	Carrying the codon-optimized gene of E6-tagged BVMO of *P. putida KT2440*	pBR322 origin, Kan^R , P_{T7}	Seo *et al.*[2]
pETDuet-E6BVMO$_{C302L}$	Carrying the codon-optimized gene of E6-tagged Cys302Leu mutated BVMO	pBR322 origin, Kan^R , P_{T7}	This study
pAPTm-E6BVMO-ADH	Carrying the codon-optimized gene of E6-tagged BVMO of *P. putida KT2440* and ADH gene of *M. luteus*	pBR322 origin, Kan^R, constitutive promoter (BBa_J23100)	Seo *et al.*[2]
pAPTm-E6BVMO$_{C302L}$-ADH	Carrying the codon-optimized gene of E6-tagged Cys302Leu mutated BVMO *P. putida KT2440* and ADH gene of *M. luteus*	pBR322 origin, Kan^R, constitutive promoter (BBa_J23100)	This study
pETDuet-E6BVMO$_{C302L\text{-}A6V}$	Carrying the codon-optimized gene of E6-tagged Cys302Leu and Ala6Val mutated BVMO	pBR322 origin, Kan^R , P_{T7}	This study
pETDuet-E6BVMO$_{C302L\text{-}A6Y}$	Carrying the codon-optimized gene of E6-tagged Cys302Leu and Ala6Tyr mutated BVMO	pBR322 origin, Kan^R , P_{T7}	This study
pETDuet-E6BVMO$_{C302L\text{-}A6W}$	Carrying the codon-optimized gene of E6-tagged	pBR322 origin, Kan^R ,	This study

	Cys302Leu and Ala6Trp mutated BVMO	P_{T7}	
pETDuet-E6BVMO$_{C302L-A6T}$	Carrying the codon-optimized gene of E6-tagged Cys302Leu and Ala6Thr mutated BVMO	pBR322 origin, Kan^R, P_{T7}	This study
pETDuet-E6BVMO$_{C302L-K483I}$	Carrying the codon-optimized gene of E6-tagged Cys302Leu and Lys483Ile mutated BVMO	pBR322 origin, Kan^R, P_{T7}	This study
pETDuet-E6BVMO$_{C302L-K483A}$	Carrying the codon-optimized gene of E6-tagged Cys302Leu and Lys483Ala mutated BVMO	pBR322 origin, Kan^R, P_{T7}	This study
pETDuet-E6BVMO$_{C302L-R31I}$	Carrying the codon-optimized gene of E6-tagged Cys302Leu and Arg31Ile mutated BVMO	pBR322 origin, Kan^R, P_{T7}	This study
pETDuet-E6BVMO$_{C302L-R31F}$	Carrying the codon-optimized gene of E6-tagged Cys302Leu and Arg31Phe mutated BVMO	pBR322 origin, Kan^R, P_{T7}	This study
pETDuet-E6BVMO$_{C302L-V21K}$	Carrying the codon-optimized gene of E6-tagged Cys302Leu and Val21Lys mutated BVMO	pBR322 origin, Kan^R, P_{T7}	This study
pETDuet-E6BVMO$_{C302L-I105K}$	Carrying the codon-optimized gene of E6-tagged Cys302Leu and Ile105Lys mutated BVMO	pBR322 origin, Kan^R, P_{T7}	This study

pETDuet-E6BVMO$_{C302L-A19K}$	Carrying the codon-optimized gene of E6-tagged Cys302Leu and Ala19Lys mutated BVMO	pBR322 origin, Kan^{R}, P_{T7}	This study
pETDuet-E6BVMO$_{C302L-D179I}$	Carrying the codon-optimized gene of E6-tagged Cys302Leu and Asp179Ile mutated BVMO	pBR322 origin, Kan^{R}, P_{T7}	This study
pETDuet-E6BVMO$_{C302L-D179F}$	Carrying the codon-optimized gene of E6-tagged Cys302Leu and Asp179Phe mutated BVMO	pBR322 origin, Kan^{R}, P_{T7}	This study
pETDuet-E6BVMO$_{C302L-L352K}$	Carrying the codon-optimized gene of E6-tagged Cys302Leu and Leu352Lys mutated BVMO	pBR322 origin, Kan^{R}, P_{T7}	This study
pETDuet-E6BVMO$_{C302L-R327I}$	Carrying the codon-optimized gene of E6-tagged Cys302Leu and Arg327Ile mutated BVMO	pBR322 origin, Kan^{R}, P_{T7}	This study
pETDuet-E6BVMO$_{C302L-R327V}$	Carrying the codon-optimized gene of E6-tagged Cys302Leu and Arg327Val mutated BVMO	pBR322 origin, Kan^{R}, P_{T7}	This study
pETDuet-E6BVMO$_{C302L-R327C}$	Carrying the codon-optimized gene of E6-tagged Cys302Leu and Arg327Cys mutated BVMO	pBR322 origin, Kan^{R}, P_{T7}	This study
pETDuet-E6BVMO$_{C302L-R327L}$	Carrying the codon-optimized gene of E6-tagged Cys302Leu and Arg327Leu	pBR322 origin, Kan^{R}, P_{T7}	This study

	mutated BVMO		
pETDuet-E6BVMO$_{C302L-R327F}$	Carrying the codon-optimized gene of E6-tagged Cys302Leu and Arg327Phe mutated BVMO	pBR322 origin, Kan^R , P_{T7}	This study
pETDuet-E6BVMO$_{C302L-R327M}$	Carrying the codon-optimized gene of E6-tagged Cys302Leu and Arg327Met mutated BVMO	pBR322 origin, Kan^R , P_{T7}	This study
pETDuet-E6BVMO$_{C302L-R327W}$	Carrying the codon-optimized gene of E6-tagged Cys302Leu and Arg327Trp mutated BVMO	pBR322 origin, Kan^R , P_{T7}	This study
pETDuet-E6BVMO$_{C302L-R327Y}$	Carrying the codon-optimized gene of E6-tagged Cys302Leu and Arg327Tyr mutated BVMO	pBR322 origin, Kan^R , P_{T7}	This study
pETDuet-E6BVMO$_{C302L-R327A}$	Carrying the codon-optimized gene of E6-tagged Cys302Leu and Arg327Ala mutated BVMO	pBR322 origin, Kan^R , P_{T7}	This study
pEKEx2-PntAB	Carrying the PntAB gene of *E. coli*	pUC18 origin, Kan^R, P_{tac}	Kabus, A. *et al.*[3]
pACYC-FadL	Carrying the FadL gene of *E. coli*	P15A origin $Chlor^R$, P_{T7}	This study

Table S2. Primers used in this study

Primer	Sequence (5’ → 3’)
P1_C302L_fw	CTGTCCGTACCTGAAGGCGACCTGTTTAAA
P2_C302L_rv	CAGACGCTGGTCCCACGGGTTATACGGCGG
P3_C302L-A6V_fw	GAAGAATCTTCTCACACCGTTCTGCCTGTTGAGCCACTC
P4_C302L-A6V_rv	GAGTGGCTCAACAGGCAGAACGGTGTGAGAAGATTCTTC
P5_C302L-A6Y_fw	GAAGAAGAATCTTCTCACACCTACCTGCCTGTTGAGCCACTCGA C
P6_C302L-A6Y_rv	GTCGAGTGGCTCAACAGGCAGGTAGGTGTGAGAAGATTCTTCTT C
P7_C302L-A6W_fw	AGAAGAATCTTCTCACACCTGGCTGCCTGTTGAGCCACTCG
P8_C302L-A6W_rv	CGAGTGGCTCAACAGGCAGCCAGGTGTGAGAAGATTCTTCT
P9_C302L-A6T_fw	GAAGAATCTTCTCACACCACCCTGCCTGTTGAGCCACTC
P10_C302L-A6T_rv	GAGTGGCTCAACAGGCAGGGTGGTGTGAGAAGATTCTTC
P11_C302L-K483I_fw	CGCGGCGCAGTGACCGATATCTGCCTGAAATTCAC
P12_C302L-K483I_rv	GTGAATTTCAGGCAGATATCGGTCACTGCGCCGCG
P13_C302L-K483A_fw	GTCGCGGCGCAGTGACCGATGCGTGCCTGAAATTCACTG
P14_C302L-K483A _rv	CAGTGAATTTCAGGCACGCATCGGTCACTGCGCCGCGAC
P15_C302L-R31I_fw	CGGCGCTGCAGCTTATCTGATCCGTAACCAGCCGAACAAAA
P16_C302L- R31I_rv	TTTTGTTCGGCTGGTTACGGATCAGATAAGCTGCAGCGCCG
P17_C302L-R31F_fw	CGGCGCTGCAGCTTATCTGTTCCGTAACCAGCCGAACAAAA
P18_C302L- R31F_rv	TTTTGTTCGGCTGGTTACGGAACAGATAAGCTGCAGCGCCG

P19_C302L-V21K_fw	CGACGTACTGATCATGGGTGCTGGTAAATCCGGTATCGGCG
P20_C302L- V21K_rv	CGCCGATACCGGATTTACCAGCACCCATGATCAGTACGTCG
P21_C302L-I105K_fw	CCAGCTGGCGCCGTTCAAACAGTACCAGCAGAAAG
P22_C302L- I105K_rv	CTTTCTGCTGGTACTGTTTGAACGGCGCCAGCTGG
P23_C302L-A19K_fw	CCACTCGACGTACTGATCATGGGTAAAGGTGTTTCCGGTATC
P24_C302L- A19K_rv	GATACCGGAAACACCTTTACCCATGATCAGTACGTCGAGTGG
P25_C302L-D179I_fw	CGCAGCACTGGCCGGAAATCCTGGACTACACCGGT
P26_C302L- D179I_rv	ACCGGTGTAGTCCAGGATTTCCGGCCAGTGCTGCG
P27_C302L- D179F_fw	CGCAGCACTGGCCGGAATTCCTGGACTACACCGGT
P28_C302L- D179F_rv	ACCGGTGTAGTCCAGGAATTCCGGCCAGTGCTGCG
P29_C302L-L352K_fw	ATCGTCACCGCGACTGGCAAAAACGTTCAGTTGTTCGGC
P30_C302L- L352K_rv	GCCGAACAACTGAACGTTTTTGCCAGTCGCGGTGACGAT
P31_C302L-R327I_fw	GATATCGTTACTGATCACATCGAAATCTTCACCGAGCACGG
P32_C302L- R327I_rv	CCGTGCTCGGTGAAGATTTCGATGTGATCAGTAACGATATC
P33_C302L-R327V_fw	CGGATATCGTTACTGATCACATCGAAGTTTTCACCGAGCACGGC
P34_C302L- R327V_rv	GCCGTGCTCGGTGAAAACTTCGATGTGATCAGTAACGATATCCG
P35_C302L-R327C_fw	TCGTTACTGATCACATCGAATGCTTCACCGAGCAC
P36_C302L- R327C_rv	GTGCTCGGTGAAGCATTCGATGTGATCAGTAACGA
P37_C302L-R327L_fw	GATATCGTTACTGATCACATCGAACTGTTCACCGAGCACGG
P38_C302L- R327L_rv	CCGTGCTCGGTGAACAGTTCGATGTGATCAGTAACGATATC

P39_C302L-R327F_fw	GATATCGTTACTGATCACATCGAATTCTTCACCGAGCACGG
P40_C302L- R327F_rv	CCGTGCTCGGTGAAGAATTCGATGTGATCAGTAACGATATC
P41_C302L-R327M_fw	CGGATATCGTTACTGATCACATCGAAATGTTCACCGAGCACGGC
P42_C302L- R327M_rv	GCCGTGCTCGGTGAACATTTCGATGTGATCAGTAACGATATCCG
P43_C302L-R327W_fw	GATATCGTTACTGATCACATCGAATGGTTCACCGAGCACGG
P44_C302L- R327W_rv	CCGTGCTCGGTGAACCATTCGATGTGATCAGTAACGATATC
P45_C302L-R327Y_fw	GATATCGTTACTGATCACATCGAATACTTCACCGAGCACGG
P46_C302L- R327Y_rv	CCGTGCTCGGTGAAGTATTCGATGTGATCAGTAACGATATC
P47_C302L-R327A_fw	GATATCGTTACTGATCACATCGAAGCGTTCACCGAGCACGG
P48_C302L- R327A_rv	CCGTGCTCGGTGAACGCTTCGATGTGATCAGTAACGATATC
P49_pACYC-FadL_fw	AGATCTCAATTGGATATCGATGGTCATGAGCCAGA
P50_pACYC-FadL_rv	TGGCCGGCCGATATCTCAGAACGCGTAGTTAAAGT

Table S3. Intramolecular interaction analysis of E6BVMO variants

E6BVMO variants	Salt bridges[a]	Hydrogen bonds[a]	Total ΔΔG (kcal/mol)[b]
C302L	R327-E330	R327-E330, R327-Q165, E330(SO)-E330(MN)[c], K483-V480, K483-Y377, K483-Y377(Ar)[d]	-
C302L/R327I	n.d.[e]	E330(SO)-E330(MN), E330-H331, H331-K342, K483-V480, K483-Y377, K483-Y377(Ar)	0.74
C302L/R327L	n.d.	E330(SO)-E330(MN), E330-H331, K483-V480, K483-Y377, K483-Y377(Ar)	0.49
C302L/R327M	n.d.	E330(SO)-E330(MN), K483-V480, H331-K342, K483-Y377, K483-Y377(Ar)	-0.21
C302L/R327A	n.d.	E330(SO)-E330(MN), E330-H331, H331-K342, K483-V480, K483-Y377, K483-Y377(Ar)	-0.36
C302L/K483A	R327-E330	R327-E330, R327-Q165, E330(SO)-E330(MN), E330-H331, K483-Y377(Ar)	0.68

[a]Salt bridges and hydrogen bonds (including Aromatic hydrogen bonds) in the vicinity of mutation residues were calculated by Maestro program of Shrödinger software package

[b]ΔΔG values were calculated by SDM software

[c]SO: side chain oxygen, MN: main chain nitrogen

[d]Ar: Aromatic hydrogen bond

[e]n.d.: not detected

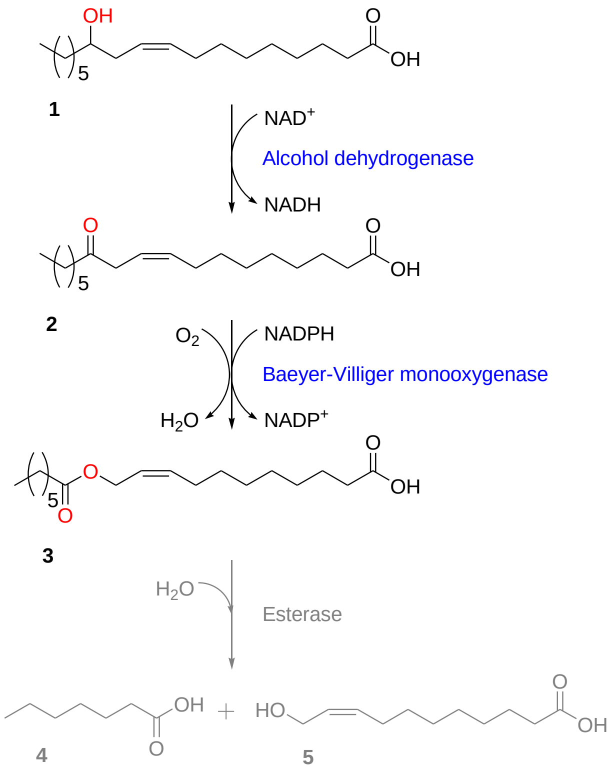

Scheme S1. Designed biotransformation pathway. Ricinoleic acid (**1**) is enzymatically converted into the ester (**3**) by an alcohol dehydrogenase and a Baeyer–Villiger monooxygenase, which can be hydrolyzed into n-heptanoic acid (**4**) and (Z)-11-hydroxyundec-9-enoic acid (**5**)[4]. Adopted from our previous study[4].

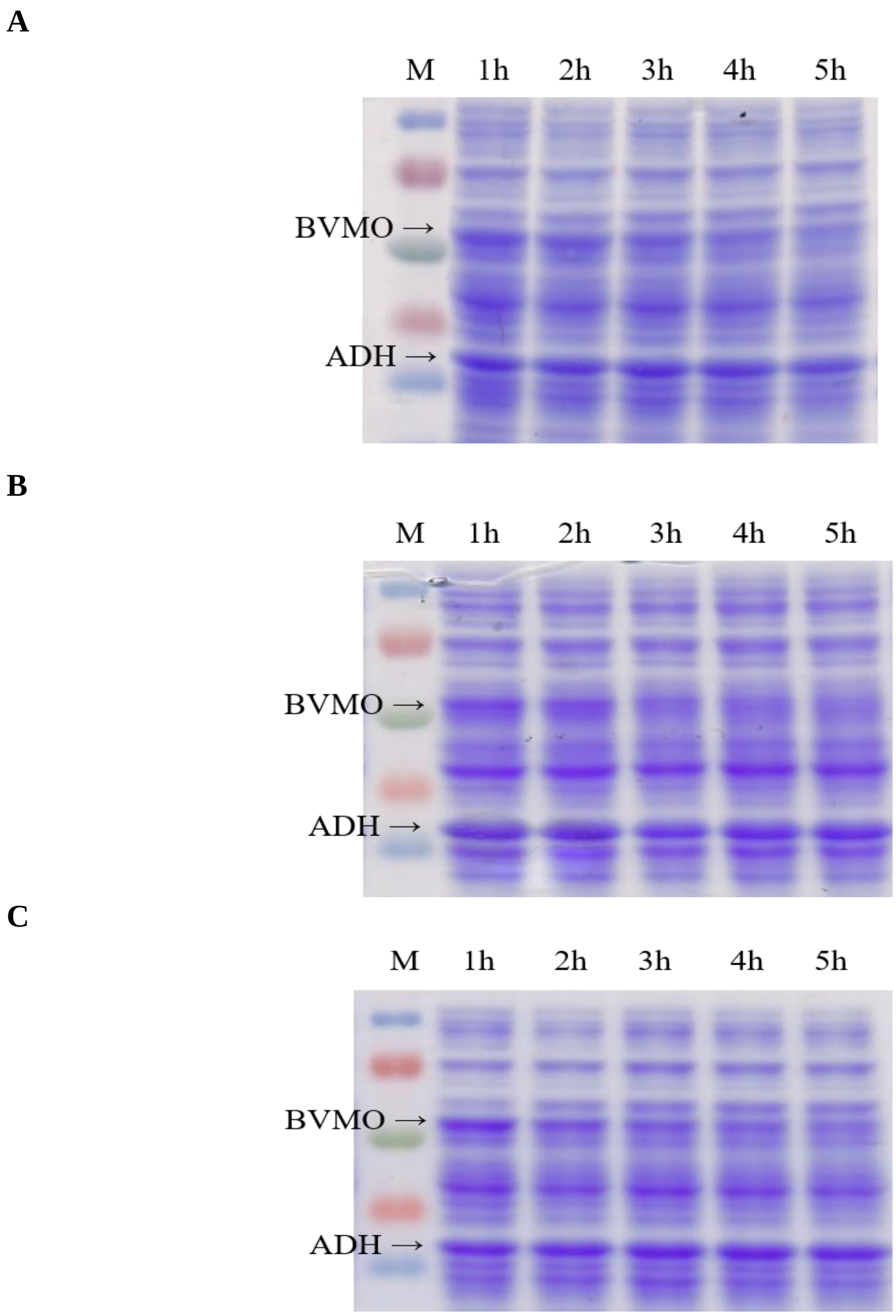

Figure S1 SDS-PAGE analysis of the protein soluble fraction changes. The soluble fraction patterns of cascade enzymes (i.e., ADH, 35 kDa and BVMOs, 55 kDa) were examined during the 15 mM ricinoleic acid biotransformation (A), incubation without ricinoleic acid (B) and incubation under microaerobic condition (C). M and numbers indicate marker and reaction or incubation time, respectively.

A

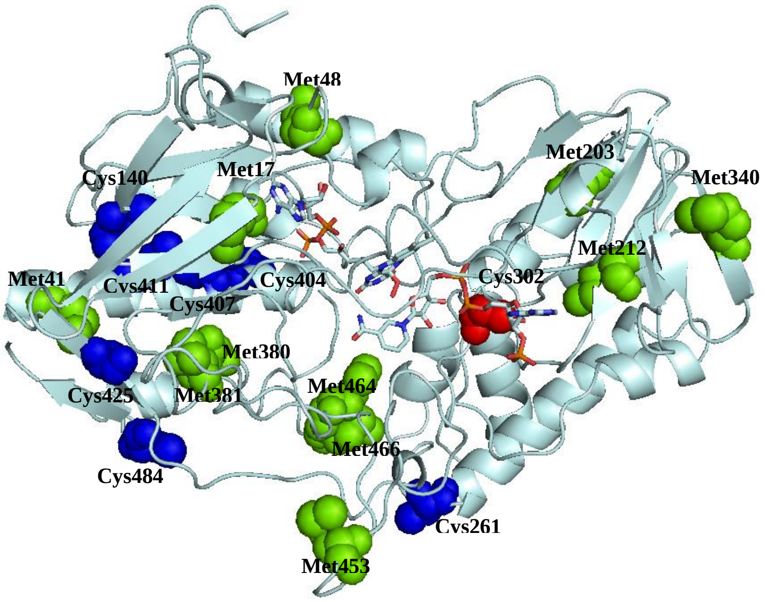

B

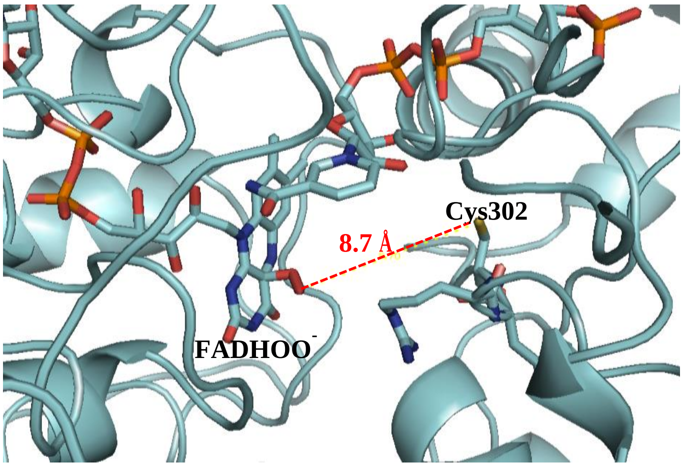

Figure S2 Ribbon diagram of the BVMO of *P. putida* KT2440, which was drawn on a basis of the previously reported homology model of the *P. putida* KT2440 BVMO[2]. Location of cysteine (blue) and methionine (green) shown as space-filled atoms (A). Cys302 which is located within the active site is shown in red. The oxygen-activating flavin-peroxide intermediate is located within 8.7 Å of the sulfur atom of the cysteine residue (B). Figures were drawn using PyMol.

A

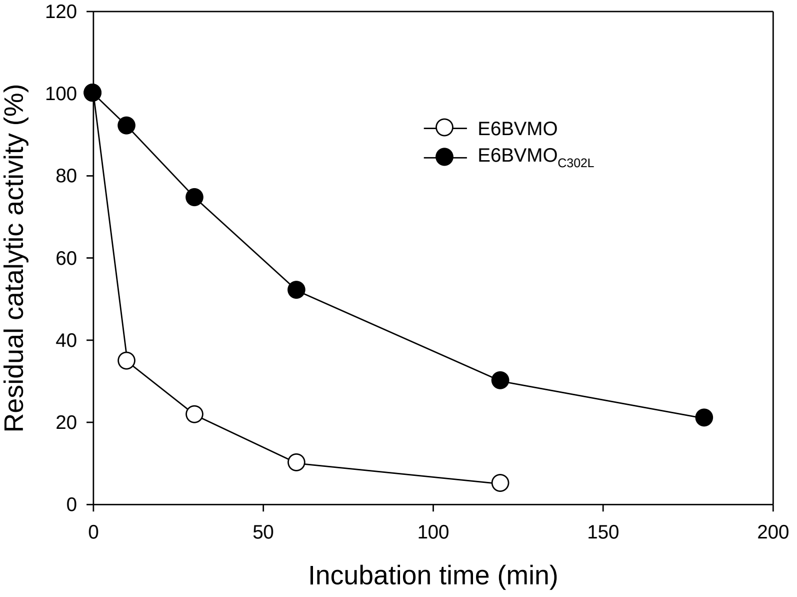

B

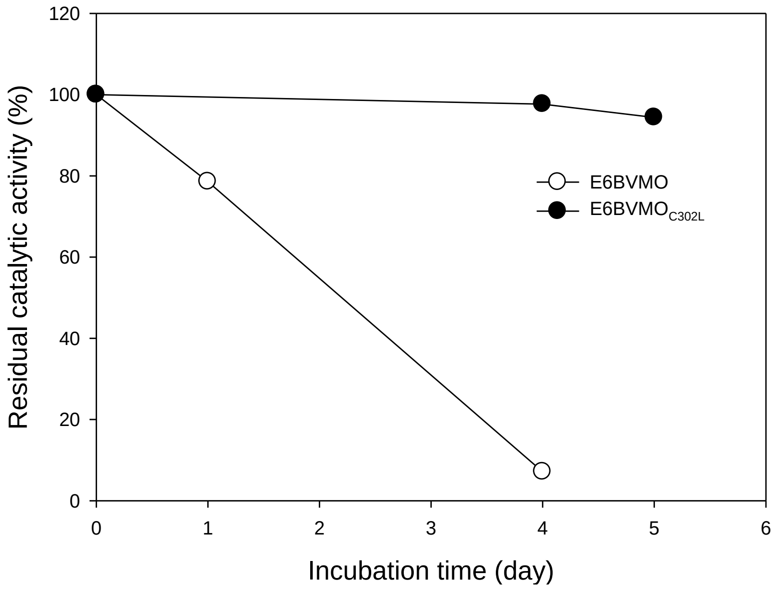

Figure S3 The stabilities of E6BVMO and E6BVMO$_{C302L}$ to thermal stresses. The residual catalytic activities of E6BVMO and E6BVMO$_{C302L}$ were evaluated at different time points during incubation at 30 (A) and 4°C (B), respectively.

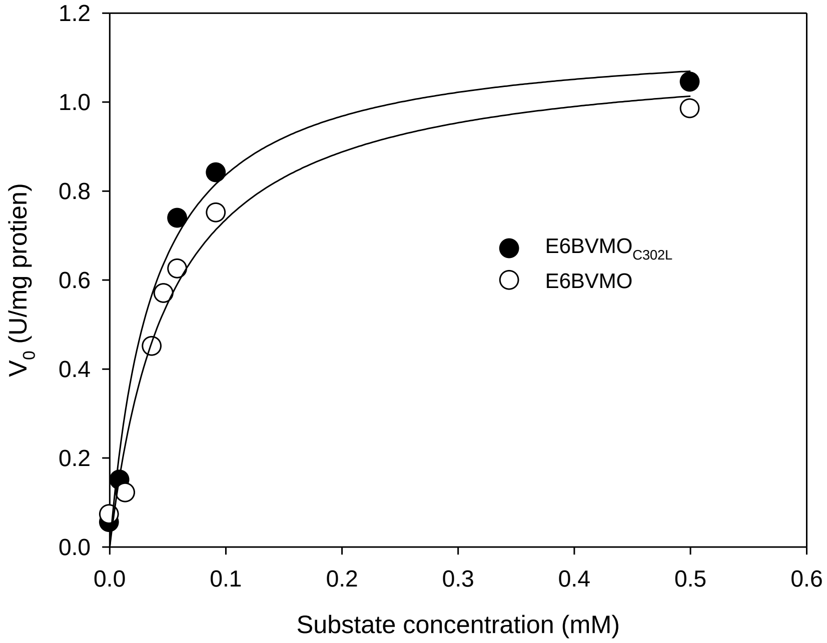

Figure S4 Enzyme kinetic plots of initial velocity versus substrate concentration of E6BVMO (open symbols) and E6BVMO$_{C302L}$ (closed symbols). The initial velocities of the engineered BVMOs were estimated by measuring the NADPH oxidation rates with 4-decanone as the substrate.

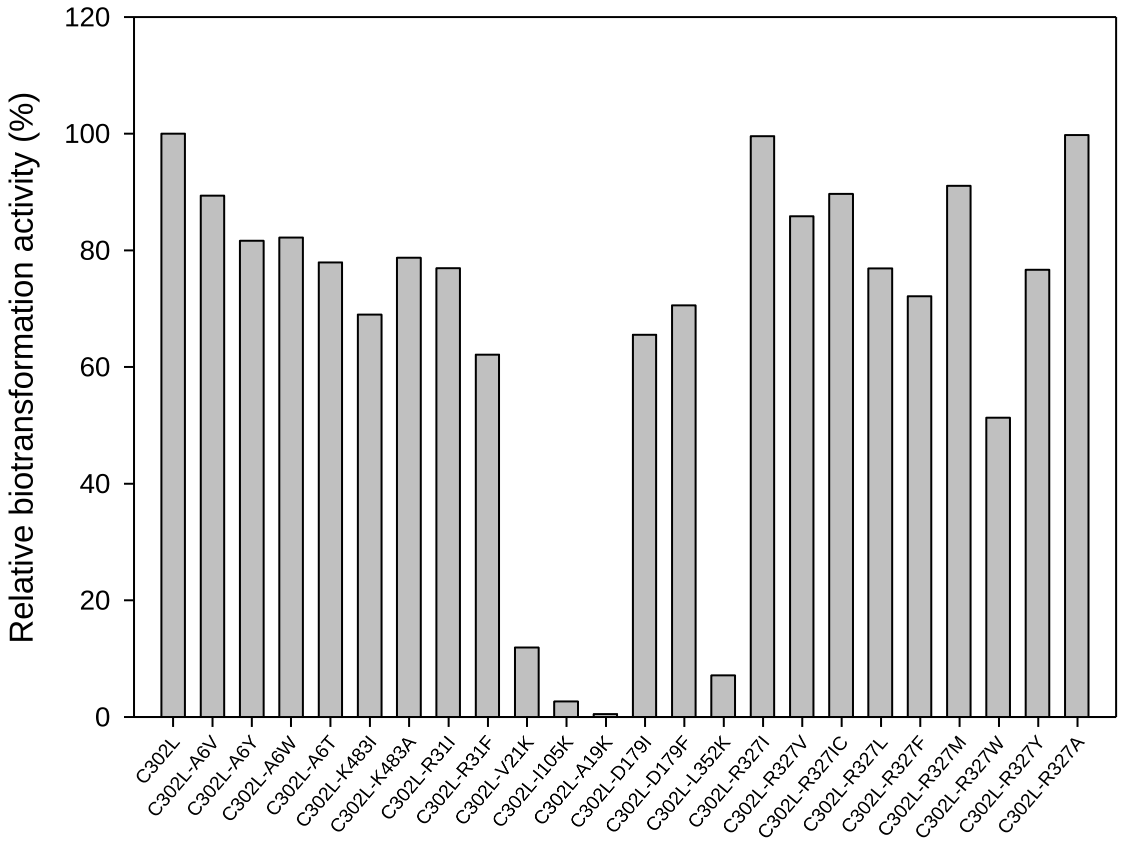

Figure S5 The biocatalytic activities of the $E6BVMO_{C302L}$ and $E6BVMO_{C302L}$-based double mutants during ricinoleic acid biotransformation reaction. The biotransformation was initiated by adding 20 mM ricinoleic acid and 0.5 g/L Tween80 into the recombinant *E. coli* culture broth (cell density: 3 g CDW/L). The biocatalytic activity was calculated based on the ester product concentration, which was determined by GC/MS, and the reaction time, which was measured when > 90% of the starting material was converted to the products.

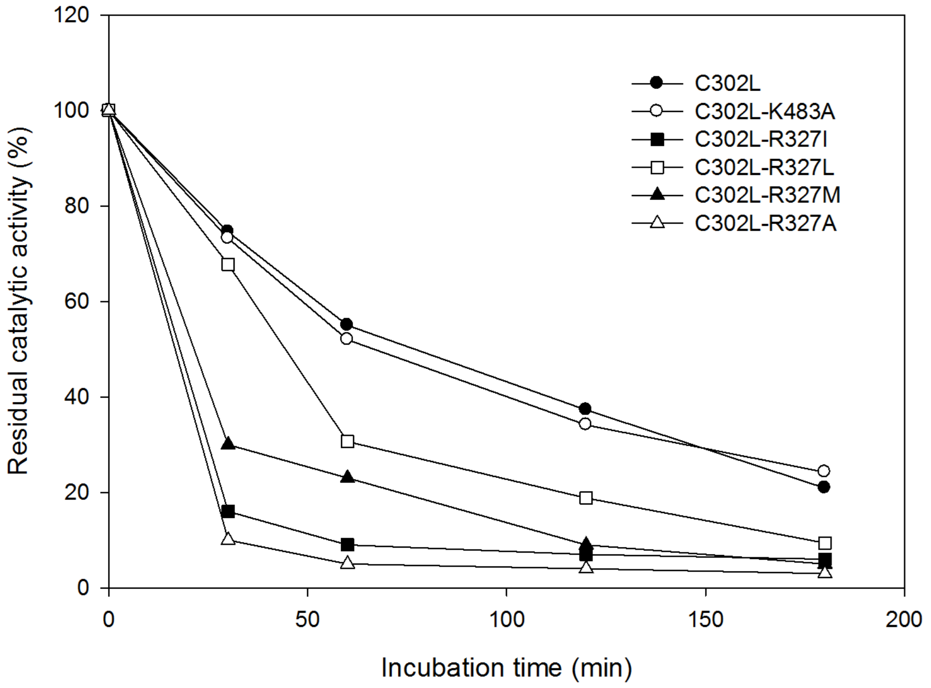

Figure S6 The stabilities of E6BVMO and E6BVMO$_{C302L}$-based double mutants to thermal stresses. The residual catalytic activities of E6BVMO and E6BVMO$_{C302L}$-based double mutants were evaluated at different time points during incubation at 30 °C.

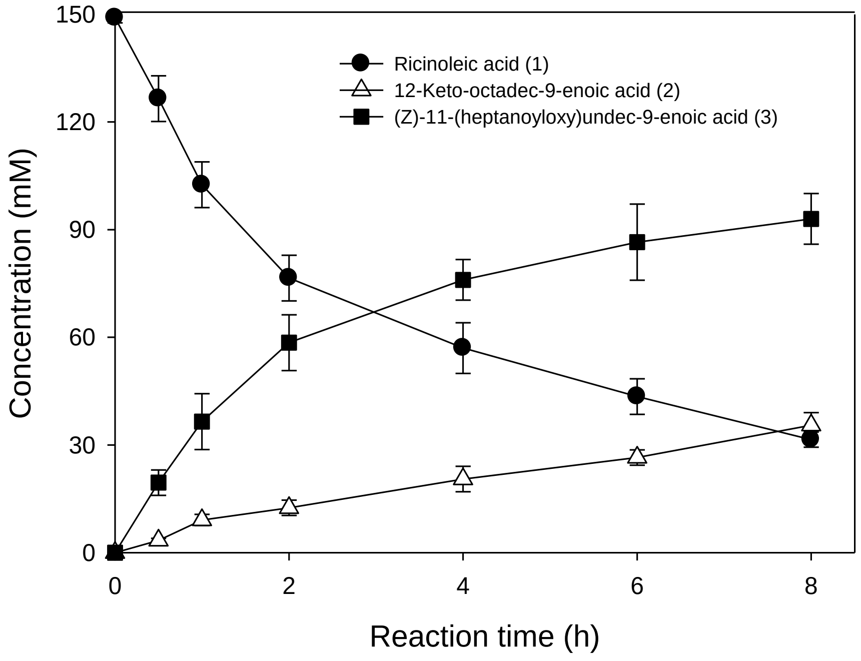

Figure S7 Time course of the biotransformation of ricinoleic acid by the recombinant *E. coli* BL21(DE3) pAPTm- E6BVMO-ADH, pACYC-FadL expressing the long chain fatty acid transporter FadL in the outer membrane in addition to the ADH and E6BVMO. The biotransformation was initiated by adding 150 mM ricinoleic acid and 0.5 g/L Tween80 into the recombinant *E. coli* culture broth (cell density: 25 g CDW/L). Symbols indicate the concentrations of ricinoleic acid (**1**) (●), 12-keto-octadec-9-enoic acid (**2**) (△), and the ester (**3**) (■).

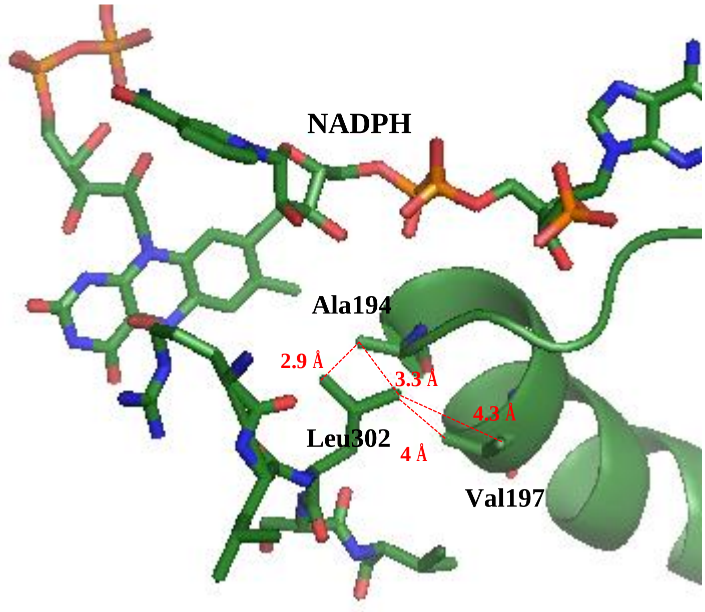

Figure S8 Model structure of the BVMO of *P. putida* KT2440 showing microenvironment of Leu302. Leu302 is located near the Ala194 and Val197. The distance between Cδ atom of Leu302 and each of Ala194 (Cβ) and Val197 (Cγ) are approximately less than 3.5 Å and 4.3 Å , respectively. Figures were drawn using PyMol.

A

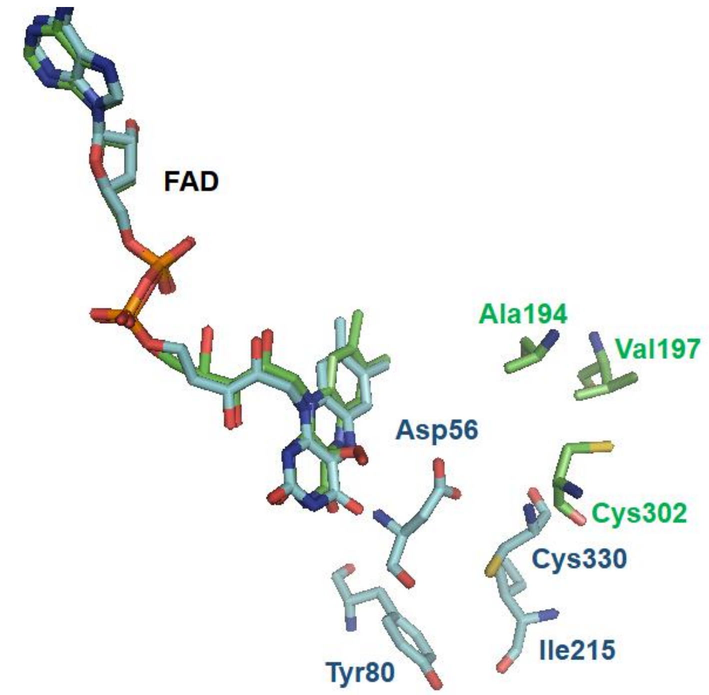
FAD
Ala194
Val197
Asp56
Cys302
Cys330
Ile215
Tyr80

B

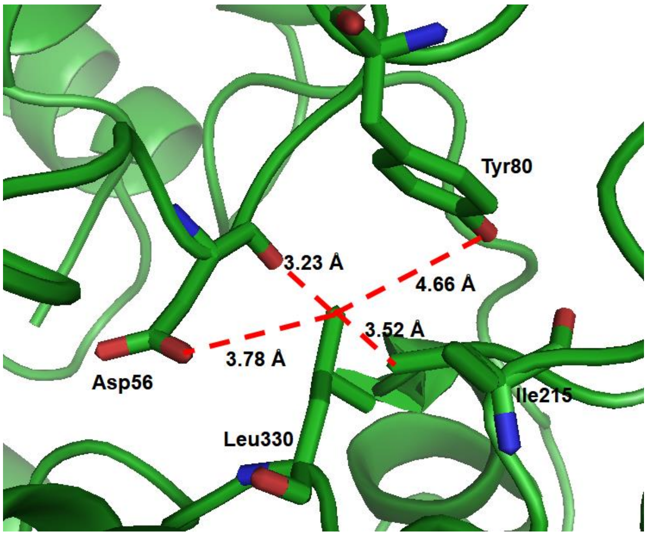
Tyr80
3.23 Å
4.66 Å
3.52 Å
3.78 Å
Asp56
Ile215
Leu330

Figure S9 The three-dimensional structure alignments of the BVMO of *P. putida* KT2440 with the cyclohexanone monooxygenase (CHMO) from *Acinetobacter* sp. NCIMB 9871[5] (A). The previously reported homology models of the *P. putida* KT2440 BVMO[2] and of the *Acinetobacter* sp. NCIMB 9871[5] were used for comparison. The green and blue letters indicated the amino acid residues from the BVMO of *P. putida* KT2440 and the CHMO, respectively. Model structure of the CHMO showing microenvironment of Leu330 (B). Leu330 is located near the Asp56, Tyr80 and Ile215. Figures were drawn using PyMol.

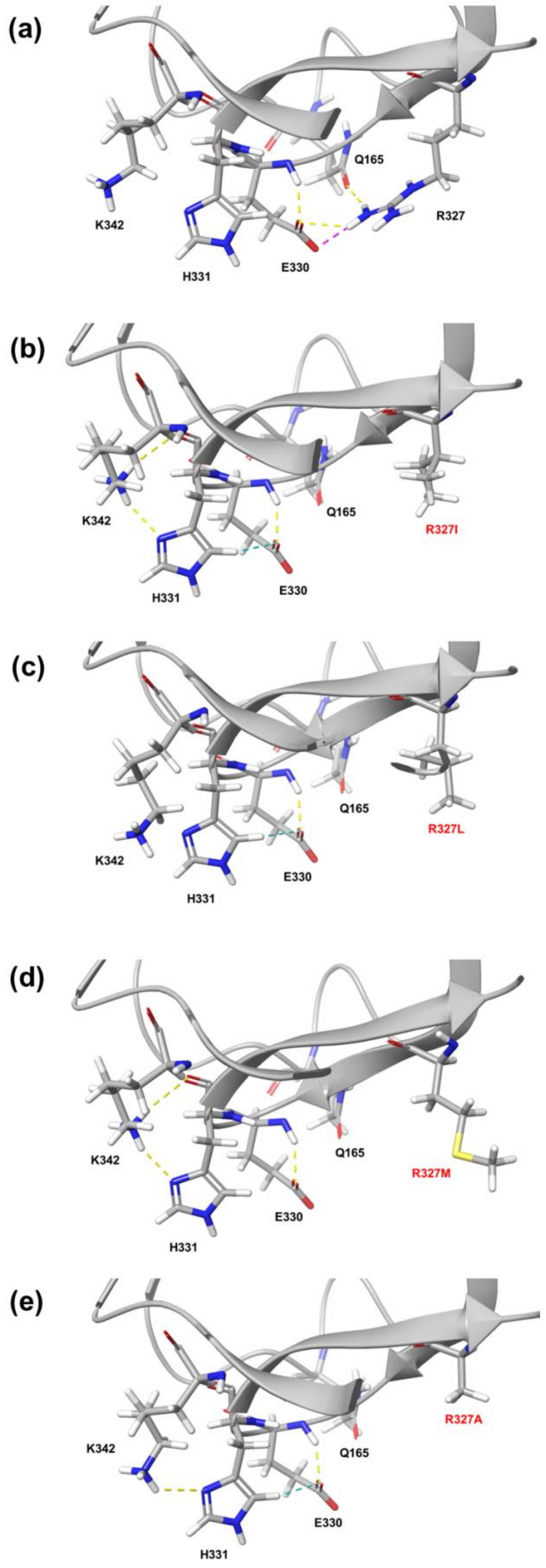

Figure S10. Intramolecular interactions of C302L mutants focusing on the R327 residue. (a) C302L, (b) C302L-R327I, (c) C302L-R327L, (d) C302L-R327M, (e) C302L-R327A; Salt

bridge (purple dotted line), Hydrogen bond (yellow dotted line), Aromatic hydrogen bond (cyan dotted line).

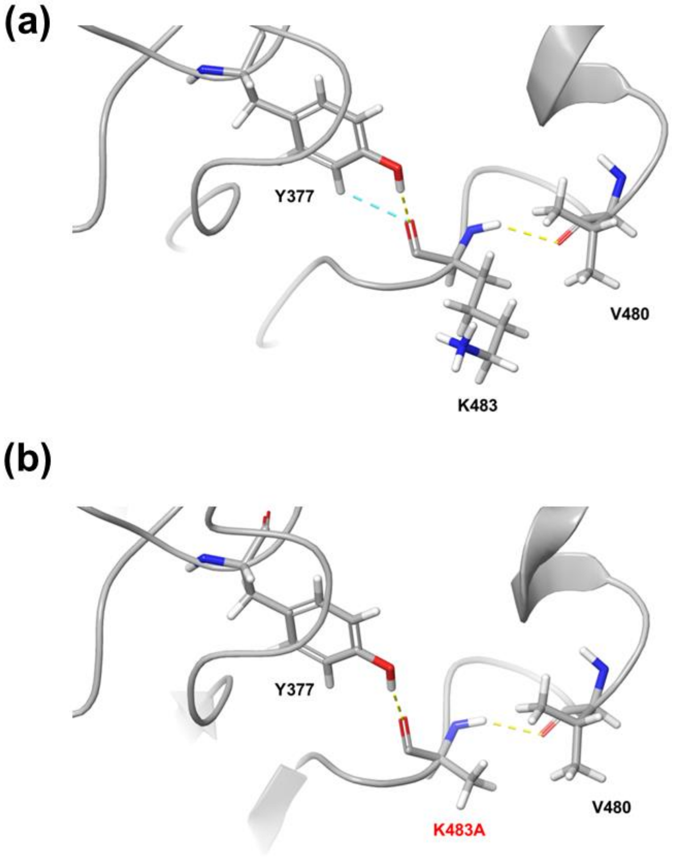

Figure S11. Intramolecular interactions of C302L mutants focusing on the K438 residue. (a) C302L, (b) C302L-K438A; Hydrogen bond (yellow dotted line), Aromatic hydrogen bond (cyan dotted line).

References

1 Song, J.-W., Lee, J.-H., Bornscheuer, U. T. & Park, J.-B. Microbial synthesis of medium-chain α,ω-dicarboxylic acids and ω-aminocarboxylic acids from renewable long-chain fatty acids. *Adv. Synth. Catal.* **356**, 1782-1788 (2014).

2 Seo, J.-H. *et al.* Engineering of Baeyer-Villiger monooxygenase-based *Escherichia coli* biocatalyst for large scale biotransformation of ricinoleic acid into (Z)-11-(heptanoyloxy)undec-9-enoic acid. *Sci. Rep.* **6**, 28223 (2016).

3 Kabus, A., Georgi, T., Wendisch, V. F. & Bott, M. Expression of the *Escherichia coli pntAB* genes encoding a membrane-bound transhydrogenase in *Corynebacterium glutamicum* improves L-lysine formation. *Appl Microbiol Biotechnol.* **75**, 47-53 (2007).

4 Song, J. W. *et al.* Multistep enzymatic synthesis of long-chain α,ω-dicarboxylic and ω -hydroxycarboxylic acids from renewable fatty acids and plant oils. *Angew. Chem. Int. Ed.* **52**, 2534-2537 (2013).

5 Opperman, D. J. & Reetz, M. T. Towards practical Baeyer-Villiger-monooxygenases: design of cyclohexanone monooxygenase mutants with enhanced oxidative stability. *ChemBioChem* **11**, 2589-2596 (2010).
